# Informal work and maternal and child health: a blind spot in public health and research

**DOI:** 10.2471/BLT.19.231258

**Published:** 2020-01-28

**Authors:** Gautam Bhan, Aditi Surie, Christiane Horwood, Richard Dobson, Laura Alfers, Anayda Portela, Nigel Rollins

**Affiliations:** a Indian Institute for Human Settlements, Bengaluru, India.; b Centre for Rural Health, University of KwaZulu-Natal, Durban, South Africa.; c Asiye e Tafuleni, Durban, South Africa.; d Women in Informal Employment: Globalizing and Organizing, Durban, South Africa.; e Department of Maternal, Newborn, Child and Adolescent Health and Ageing, World Health Organization, 20 avenue Appia, 1211 Geneva 27, Switzerland.

More than 2 billion people (about 61% of the global workforce) are engaged in the informal economy; this represents 88% of total employment in India, over 80% for countries as diverse as the Plurinational State of Bolivia, Ghana, Indonesia and Morocco, and even 19% in the United States of America.^1^ In many low- and middle-income countries, informal work is the rule and not the exception. Informal economy workers cross a range of sectors, the most common being street vending, domestic work, waste picking, home-based work (such as producing garments or handcrafts) and construction. For these workers, caring for themselves and their children presents unique challenges. Mothers who work in the informal sector must continue to bring income to the household, care for their physical and mental health after childbirth, and attempt to exclusively breastfeed their infant and provide nurturing care. They must do all of this while working without any formal labour protection, such as maternity leave. In the informal economy, there are few, if any, public or private social protection initiatives to facilitate access to health care, protect income security or mitigate risks that help reconcile the tension between being a worker, a woman and a mother. Remarkably, public health research and practice has so far largely ignored this group. 

How do women working in the informal economy manage care for themselves and their young children while earning a sufficient income without any of the benefits usually associated with formal employment? Here we briefly describe the scale and importance of recognizing informal employment from a health perspective and consider pathways to alleviating the trade-off that mothers working in the informal sector face. As an illustration, we explore the difficulties for mothers wanting to exclusively breastfeed their infants during the first six months, as recommended by the World Health Organization (WHO), while still working in the informal economy.

## Informal employment and health

Informal workers generally do not enjoy minimum wages, maternity (or paternity) leave, job and wage security and predictability or occupational health and safety. Their work is not recognized (home-based workers are rarely counted, for example, in official statistics), is marginalized, and often even criminalized (for instance through laws against street vending or waste picking). Thus, while informal work is testament to the productive capacities of workers, it also is vulnerable, low-quality and precarious, and lacks access to legal protection, modern capital markets, formal training and official social security systems.^2^ These aspects are also possible explanations as to why informal work has remained outside the research and policy agendas of global public health.

In China, Latin America and sub-Saharan Africa, nearly half of workers in informal employment are women, as are a fifth in South-east Asia.^3^ Therefore, globally, women’s informal work is a central feature of the feminization of poverty and a core pathway for progress towards the sustainable development goals (SDGs), particularly those related to health (SDG 3), gender equality (SDG 5) and decent work and economic growth (SDG 8). Despite these facts, we know little about how informal work affects maternal, newborn and child health. How do women working in the informal economy cope with the effects of economic vulnerability and difficult working conditions and how does this vulnerability affect their ability to attain a secure income? How does it affect early infant care practices, such as breastfeeding, and what are the longer-term effects on the health and development of children?

Such workers are the one low-income group that is particularly neglected when it comes to health.^4^ A seven-part *Lancet* Series on universal health care in India^5^ referred to the informal sector only twice, despite macro-empirical studies that indicate labour market inequalities have robust co-relations with health outcomes.^6^ Importantly, informal workers perceive that their work has a negative impact on their health.^7^ WHO’s *A conceptual framework for action on the social determinants of health*^8^ does mention income and the physical conditions of workplaces, but not of the broader conditions of employment including informal employment, nor the existence of contracts, rights and access to social protection.

**Fig. 1:**
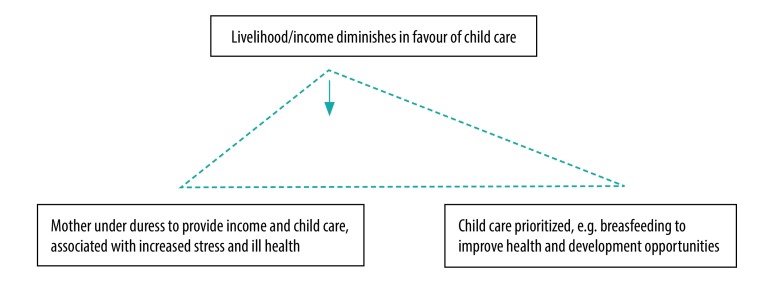
Competing demands on mothers working in the informal economy

Note: The figure shows a scenario where livelihood income may diminish if childcare is prioritized.

Furthermore, international guidelines for improving child health, such as promoting exclusive breastfeeding may result in competing priorities and expectations, adding to the complexity and stress of these women’s lives (Fig. 1).

The interaction between informal livelihood, maternal health and infant care is not substantively reflected in public health thinking. The lack of descriptive peer-reviewed reports and intervention studies highlights that research examining the significance of relationships between informal work, social protection and maternal and child health outcomes is long overdue. Such research must look at the impact of conditions and quality of work as well as women’s ability to earn secure incomes and to address child care. Research is needed to identify constructive interventions, social protection and health service delivery innovations, and policy responses are required to provide stability, expand opportunities and reduce vulnerability in this period of a working woman’s life.^9^ Only a few formative studies have examined the relationships between conditions and quality of work and maternal and child health. Findings from street vendors, domestic workers and home-based workers in New Delhi, India, and Durban, South Africa, suggest possible pathways for intervention that future research needs to test and validate.^10^

## Pathways to impact

Three characteristics of these women’s lives exemplify the links between informal work, social protection and maternal and child health, and offer opportunities to modify urban ecosystems in favour of mothers and children.

First, informal workers are much more likely to return to work soon after childbirth because of the absence of an employment contract and its associated entitlements. Without structured maternity leave and compensatory income, mothers, in Durban and Delhi, return as early as three weeks after childbirth and most are back at work in less than two months. Work insecurity and unpredictability, the fear of losing a vending spot, for example, add to these pressures and deter or delay care-seeking for both mothers and their infants. As women resume work, workplace conditions (a sidewalk for street vendors or a private home for domestic workers) often hinder the proximity between mother and infant that is needed for breastfeeding.

Second, multiple overlapping dimensions of income poverty, informal work and socioeconomic marginalization are likely to reinforce each other to adversely affect the health of mothers, their opportunity to care for their infants and the health and development of their children. Many women earn variable amounts daily, with few options to withdraw from work or change working conditions after childbirth. In India and South Africa, women’s income is often the only or primary income of the household. Women working in the informal sector also tend to be members of socially marginal racial, religious, and in the case of India, caste groups. Studies report that such households are more prone to welfare and health shocks (injury, natural disasters, unemployment or hospitalization) and to costly coping strategies, such as expensive loans, or reducing consumption expenditure (such as on fuel and food) or sending children to work.^11^

Third, our work indicated high levels of food insecurity, variable and inconsistent childcare arrangements and related anxiety and stress.^10^ These stresses emanate from sociopsychological dilemmas for these working mothers beyond the pressures of low income or newborn care. Many women spoke of feeling torn between working and being mothers, expressed anxiety about managing domestic and work pressures, and struggled with a sense that that they were not good mothers. While these debates on the double shift of women’s work are not new, they are exacerbated within the context of informal work and manifest differently than in formal work.

## Future research

Seeing women as individuals with their own lives rather than as workers or as mothers, challenges current paradigms for research and policy to protect and promote maternal, newborn and child health and development.

Research is needed to understand the interactions between employment conditions (economic relationships, social protection, labour rights and work benefits), workplace conditions (general physical and psychosocial conditions of work) and health outcomes of women, pregnant women and mothers, as well as their children. WHO’s conceptual framework must take on the specifics of diverse forms of work. Experimentation and innovation must challenge the traditional models of social protection that conventionally depend on employers or the workplace to deliver benefits.

**Fig. 2:**
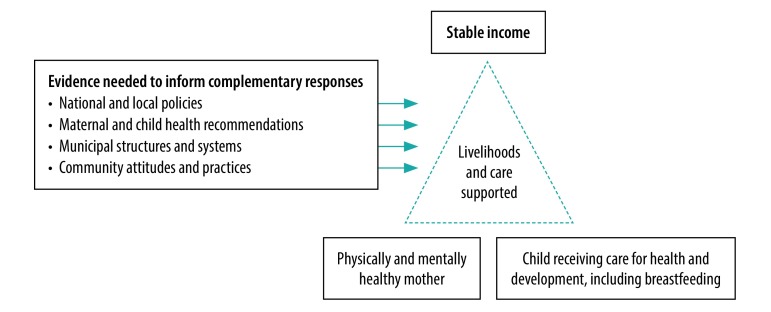
Research areas to support the livelihood, health and development of mothers working in the informal economy and their children

This research must be inter-disciplinary and comparative to enable some generalizability of findings and to assemble international recommendations, guide national policy and inform responses among city planners and health authorities (Fig. 2). This knowledge is essential if the International Labour Organization’s (ILO) centennial goals towards the Future of Work^12^ (a re-framing of the Women at Work initiative including maternity protection) and if the SDGs are to be fulfilled. Such a research enterprise would be opportune given recent ILO recommendations that speak of addressing the specific vulnerabilities of informal work and the urgency to remedy them. Attempts to find appropriate labour regulations for different sectors of informal work are emerging and will need to be informed to include working mothers’ health in addition to their rights and earnings as workers. Such labour regulations would create enabling conditions for working mothers to care for themselves and facilitate their children reaching their health and developmental potential.
